# Information technology-based joint preoperative assessment, risk stratification and its impact on patient management, perioperative outcome, and cost

**DOI:** 10.15190/d.2021.9

**Published:** 2021-06-30

**Authors:** Habib Md Reazaul Karim, Subrata Kumar Singha, Praveen Kumar Neema, Tridip Dutta Baruah, Rubik Ray, Debajyoti Mohanty, Md Sabah Siddiqui, Rachita Nanda, Narendra Kuber Bodhey

**Affiliations:** ^1^Department of Anaesthesiology, India Institute of Medical Sciences, Raipur, India; ^2^Department of General Surgery, India Institute of Medical Sciences, Raipur, India; ^3^Department of General Medicine, India Institute of Medical Sciences, Raipur, India; ^4^Department of Clinical Biochemistry, India Institute of Medical Sciences, Raipur, India; ^5^Department of Radiodiagnosis, India Institute of Medical Sciences, Raipur, India

**Keywords:** Information technology, software, preoperative care, elective surgical procedures, health resources, cost-benefit analysis.

## Abstract

Background: Despite negative recommendations, routine preoperative testing practice is nearly universal. Our aim is to bring the healthcare providers on one platform by using information-technology based preanaesthetic assessment and evaluate the routine preoperative testing’s impact on patient outcome and cost. Methods: A prospective, non-randomised study was conducted in a teaching hospital during January 2019-August 2020. A locally developed software and cloud-computing were used as a tool to modify preanaesthesia evaluation. The number of investigations ordered, time taken, cost incurred, were compared with the routine practice. Further data were matched as per surgical invasiveness and the patient's physical status. Appropriate tests compared intergroup differences and p-value <0.05 was considered significant. Results: Data from 114 patients (58 in routine and 56 in patient and surgery specific) were analysed. Patient and surgery specific investigation led to a reduction in the investigations by 80-90%, hospital visit by 50%, and the total cost by 80%, without increasing the day of surgery cancellation or complications. Conclusion: Information technology-based joint preoperative assessment and risk stratification are feasible through locally developed software with minimal cost. It helps in applying patient and surgery specific investigation, reducing the number of tests, hospital visit, and cost, without adversely affecting the perioperative outcome. The application of the modified method will help in cost-effective, yet quality and safe perioperative healthcare delivery. It will also benefit the public from both service and economic perspective.

## INTRODUCTION

Healthcare costs are continually escalating worldwide, making healthcare providers and authorities concerned and target-oriented in cost-effective, yet of quality and safe healthcare delivery. Surgical care cost is a considerable amount, and the usefulness of routine preoperative testing as a part of preoperative assessment has been rigorously scrutinised^1^. The American Society of Anesthesiologists (ASA) and the National Institute of Clinical and Health Excellence (NICE) have been guiding the use of preoperative tests before elective surgeries for the last one and a half-decade^2,3^. The currently available evidence recommends against the practice of 'routine preoperative laboratory tests' and recommends patient and surgery-specific investigations. Despite having these negative recommendations, the tradition is still prevalent and does not follow the guidelines^4,5^. The reasons are multitudinal^6^. One of the reasons is the belief that other persons involved, i.e., physicians/ anaesthesiologists, want the tests to be performed^6^. Bringing the anaesthesiologists and surgeons on the common platform will improve communication and may nullify these reasons. It is also shown that many anaesthesiologists and surgeons believe that preoperative investigations should be patient and surgery specific^6^. We hypothesise that information technology-based (IT-based) joint preoperative assessment might reduce unnecessary laboratory tests, decrease hospital visits, and save costs. In everyday practice, the surgeon initially examines the patients and orders the investigations. Mainly, tests are ordered as routine testing and diagnostic. Contrarily, sometimes required testing or evaluations are also left out, which affects the patients^7^. We conducted this study to assess and compare the preoperative tests ordered before and after implementing an IT-based module with cost analysis. The secondary aim was to compare the perioperative outcome of the surgery and safety and pitfalls (if any) of patient and surgery specific investigations and routine investigation in perioperative management.

## METHODS AND SUBJECTS

We performed a prospective, non-randomised, clinical study with a non-parallel, non-crossover design, with non-concurrent control recruitment. This study was conducted from January 2019 to August 2020, in a teaching institute. The study was approved by the institute research and ethical committee and registered with www.ctri.in (CTRI/2018/11/016441). The Helsinki declaration and good clinical practice were followed. Informed written consent was obtained from patients above 18 years old, and the parents or legal guardians and the adolescent's assent for all participants of 16-18 years of age.

Patient recruitment took place in two phases. In the first phase, pre-anaesthetic evaluation was conducted as per the existing institutional standard operating procedure, where a patient usually attends the pre-anaesthesia evaluation clinic (PAEC) with routine investigations done. No intervention was done to modify or change the evaluation procedure. The research assistant screened all patients attending PAEC, and those who fulfilled the inclusion and not falling into exclusion criteria were approached for consent after explaining the purpose of the study. A patient information sheet in both English and Hindi was available to hand over to the patient. Patients planned for elective non-cardiac surgery, aged more than or equal to 16 years of either male or female belonging to the ASA-physical status (ASA-PS) I to III were included. Pregnant and lactating women, emergency surgeries, and ASA-PS IV and above were excluded. The project research assistant filled the form in a hard copy once the PAEC was over and the case was cleared for elective surgery.

**Figure 1:**
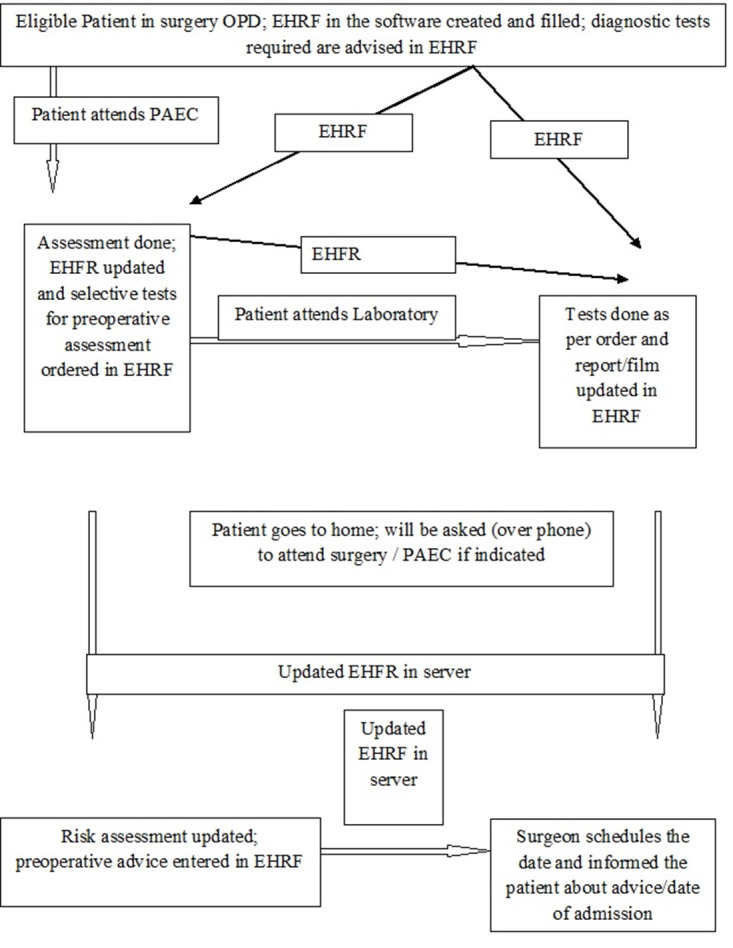
Schematic representation of the proposed workflow for joint preoperative assessment and patient management EHRF - electronic health record file, OPD - outpatient department, PAEC - preanaesthesia evaluation clinic.

In the second phase, a software (Perioperative Anaesthesia Data Management Software©) was developed for Windows using C-sharp language and was installed in the various computers of PAEC, faculty rooms, operation theatre, laptop of research assistant and the principal investigator. The computers were linked through cloud-computing technology. The research assistant entered the data, and the data entry software was secured using a username and password for users, with the principal investigator having administrator capability. Once the data entry was over, the patient's particular case record form was locked, and only the administrator could unlock or edit the data further. Post-locking, the data was available as read-only, printed in .pdf format, and exported in Microsoft Excel. The eligibility assessment, consent, and recruitment criteria were the same, but the preanesthetic evaluation was modified, and the patient was evaluated both by the surgeon and anaesthesiologists before ordering the investigations. The investigations ordered were patient and surgery specific investigations. For this purpose, a published guide table formulated from nearly ten guidelines and recommendations were used to decide the investigation^4^. The surgeon and the anaesthesiologist also discussed any relevant information over the phone when required. The IT-based joint preoperative assessment and risk stratification model workflow was adapted from a concept^8^ and it is presented in the Figure 1.

The sample size was calculated based on a previously conducted Indian study, where the prevalence of routine preoperative investigations was nearly 99%^4^. We assumed a reduction of 25% in preoperative testing prevalence (i.e., unexposed routine group 99%; patient and surgery specific group 75%). The two-sided significance (1 – α) of 95% and power (1 – β) of 80% was taken. Online epidemiological tool OpenEpi (Open-Source Epidemiologic Statistics for Public Health; http://www.openepi.com/) was used for calculating the sample (Fleiss method with continuity correction). Taking exposed to the non-exposed ratio of 1:1 and a design effect of 1.4 to compensate for non-randomised design, along with 10% dropouts, the final sample required was a minimum of 58 participants per group (total 116 at minimum).

Data in the aspects of demographic and clinical characteristics, vital clinical parameters, physical status, NICE surgical grade of invasiveness, number of investigation done, number of hospital visits by the patients till the patient is cleared for surgery, delay (if any), a perioperative outcome like high-dependant / intensive care stay, delayed awakening or any morbidity, were noted in the case record form/software. Intraoperative and postoperative instances where it was felt that the routine preoperative test would have contributed to decision making and management was taken as pitfall/deficiency of patient and surgery specific investigations. Reasons/background of such instances, if any, were also noted in the remarks section.

Phase I data (Group A - routine investigation) were entered manually in the Microsoft Excel master chart. The phase II data (Group B - patient and surgery specific investigations) were exported directly as Excel sheets and re-verified for typographical errors and obvious mistakes and discrepancies. The research assistant prepared the master chart and did necessary calculations without interference, except for correcting ASA-PS class and NICE surgical grades, if required from the history and clinical findings available. The analysis was performed both for the entire cohort and ASA-PS and NICE grade matched (1:1 categorical matched) cohorts. Qualitative data are calculated and presented in absolute numbers and percentage scale, whereas discrete quantitative data are presented in numbers and median and interquartile ranges (q3-q1). Fisher's exact test was used for assessing the intergroup differences. Quantitative continuous data are presented in mean + standard deviation (SD). The Kruskal-Wallis test or unpaired t-test was used to compare intergroup differences based on the data distribution pattern tested with the k-test. The cost was calculated in terms of Indian rupees, and reduction is presented on a percentage scale. Statistical analysis of the data was done using INSTAT software (GraphPad Software, Inc, La Jolla, CA, United States), and two-tailed p<0.05 were considered significant.

## RESULTS

**Figure 2:**
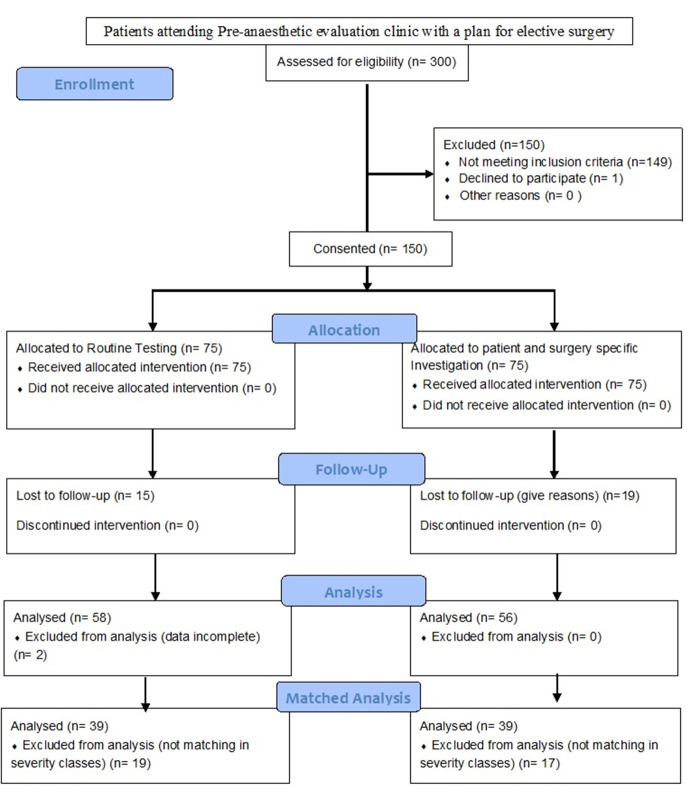
Flow diagram of the participants n-number.

A total of 150 (75 each) participants were recruited. While sixty patients in the routine group could be followed up until the study, only 56 participants could complete the study in the patient and surgery specific group, and due to the impact of the Coronavirus disease of 2019 (COVID-19) pandemic on elective cases in our institute, the study was ended with permission. Two participants were excluded from analysis for incomplete data from the routine group, and a final sample of 58 and 56 were included for analysis in the routine and patient and surgery specific group, respectively. The participants were then matched 1:1 for both ASA-PS and NICE grades, and a total of 39 participants per group were compared. The study result is reported as per STROBE guideline, and the flow diagram is presented in Figure 2.

Patients were distributed almost equally in terms of gender. The mean age, weight, height, body mass index, and the metabolic equivalent of tasks categorical distributions were also statistically indifferent. Although the number of ASA-PS class I patients was higher in the patient and surgery specific group (53.6% versus 43.1%), the difference was statistically insignificant; p=0.348. However, the NICE surgical invasiveness grades' differences were statistically significant for class 1 and 2 between the groups; p=0.001 for both (Table 1).

Except for the HbA1c, the number of tests (complete blood count, blood sugar tests, serum electrolytes, blood urea, serum creatinine, electrocardiogram, chest x-ray, coagulation profile, liver function, and thyroid function tests) done was significantly higher in the routine group; p<0.0001 for the most (Table 2).

The difference persisted when ASA-PS and NICE grade matched groups were compared, except for CBC and thyroid function tests (Table 3).

Nearly 85% in the routine group and 91% of the patient and surgery specific group patients were cleared in one visit, p=0.394. No difference was noted in the matched groups (Table 4).

**Figure 3:**
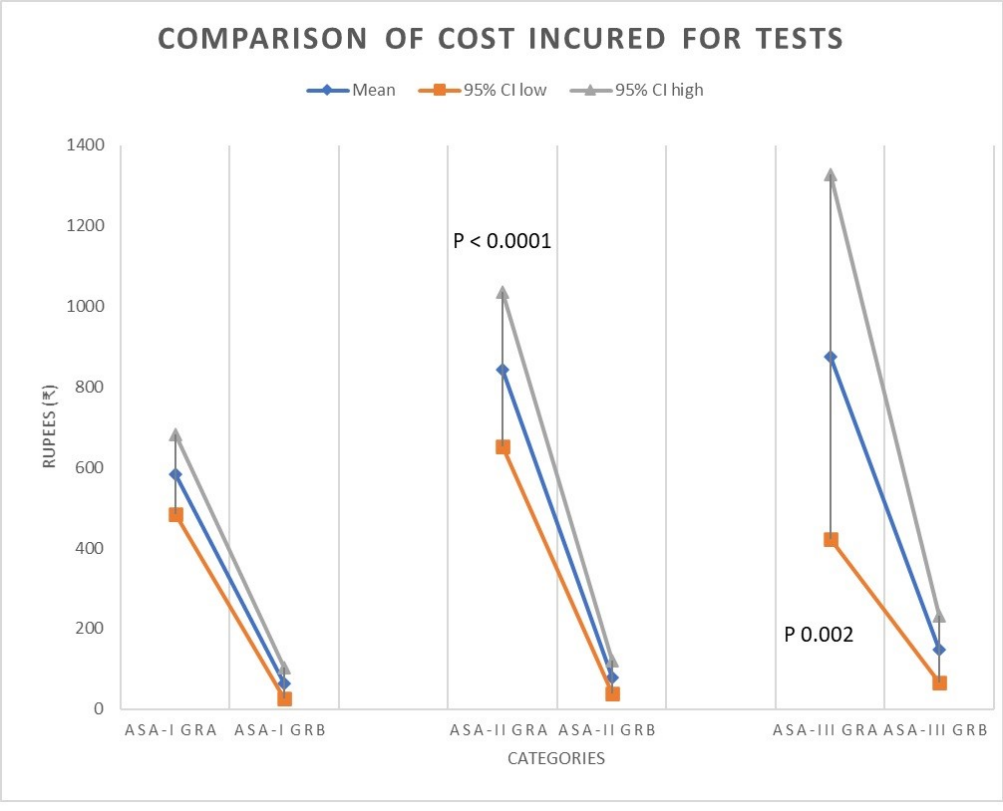
Cost reduction across the different ASA-PS classes (categories) Group A - routine testing, group B - patient and surgery specific testing; ASA-PS - The American Society of Anesthesiologists physical status class. The group A represents routine investigation and group B represents surgery and patient specific investigation.

There was a 50% reduction in the number of hospital visits in the patient and surgery specific group than the routine group, p<0.0001, and 80 to 90% reduction in the cost for travel, tests, and total expenditure, p<0.0001 for all (Table 5). The difference held even when ASA-PS and NICE grade matched groups were compared (Table 6).The mean preoperative investigation costs increased for the ASA-PS I, II, and III in both groups. There was a reduction of the cost incurred by Indian rupees 518 (89.0%), 764 (90.6%), 726 (83.1%) respectively for ASA-PS I, II, and III patients, and the differences were extremely significant. Figure 3 presents the mean and 95% confidence level of the costs for tests.

**Table 1:** Clinico-demographic distribution of the cohorts compared using unpaired t-test and Fisher’s exact test ASA - American Society of Anesthesiologists, NICE - National Institute for Health and Clinical Excellence, METs - Metabolic Equivalents for Tasks, N - total number). Group A represents routine investigation, and group B represents surgery and patient specific investigation.

Parameter	Group A [N=58]	Group B [N=56]	Two-sided p-value
Male	31(53.44)	33(58.92)	0.576
Female	27(46.55)	23(41.07)	
Age (years)	40.81±14.60	39.39±13.47	0.591
Weight (kilogram)	61.53±13.50	61.36±13.87	0.947
Height(centimeter)	157.82±19.62	159.54±10.44	0.562
Body Mass Index (kg/m2)	24.19±4.41	24.03±4.56	0.854
ASA - I	25(43.10)	30(53.57)	0.348
ASA - II	30(51.72)	21(37.5)	0.136
ASA – III	03(5.17)	05(8.92)	0.486
NICE surgical grade 1	10(17.24)	26(46.42)	0.001
NICE surgical grade 2	26(44.82)	09(16.07)	0.001
NICE surgical grade 3	21(36.20)	19(33.92)	0.846
NICE surgical grade 4	01(1.72)	02(3.57)	0.615
METs 0-4 (low)	01(1.72)	02(3.57)	0.615
METs 5-9 (intermediate)	56(96.55)	52(92.85)	0.434
METs 10 (good)	01(1.72)	02(3.57)	0.615

**Table 2:** Comparison of the total number of tests done among the cohorts tested using Fisher’s exact test LFT - liver function tests, RFT - renal function tests, PT - prothrombin time, INR - international normalized ratio, APTT - activated partial thromboplastin time, HbA1c - glycosylated hemoglobin, TFT - thyroid function test, CXR - chest x-ray, ECG - electrocardiogram. Group A represents routine investigation, and group B represents surgery and patient specific investigation.

Parameter (tests)	Group A [N=58]	Group B [N=56]	Two-sided p-value
CBC	57(98.27)	47(83.92)	0.007
Blood Sugar	32(55.17)	5(8.9)	<0.0001
LFT	50(86.20)	0	<0.0001
RFT	52(89.65)	5 (8.9)	<0.0001
PT/INR, APTT	20(34.48)	0	<0.0001
HbA1C	02(3.44)	0	0.495
TFT	06(10.34)	0	0.027
Serum Electrolyte	57 (98.27)	09(16.1)	<0.0001
CXR	22(37.93)	0	<0.0001
ECG	28(48.27)	06(10.6)	<0.0001

**Table 3:** Comparison of the total tests performed in matched cohorts tested using Fisher’s exact test ASA - American Society of Anesthesiologists, NICE - National Institute for Health and Clinical Excellence, N - Total number, LFT - liver function tests, RFT - renal function tests, PT - prothrombin time, INR - international normalized ratio, APTT - activated partial thromboplastin time, HbA1c - glycosylated hemoglobin, TFT - thyroid function test, CXR - chest x-ray, ECG - electrocardiogram). Group A represents routine investigation, and group B represents surgery and patient specific investigation.

Parameters/ tests		ASA-PS matched			NICE grade matched	
	Group A [N=39]	Group B [N=39]	Two-sided p-value	Group A [N=39]	Group B [N=39]	Two-sided p-value
CBC	38(97.43)	35(89.74)	0.358	38(97.43)	34(87.17)	0.200
Blood Sugar	24(61.53)	04(10.25)	<0.0001	23(58.97)	04(10.25)	<0.0001
LFT	33(84.61)	0	<0.0001	34(87.17)	0	<0.0001
RFT	34(87.17)	04(10.25)	<0.0001	34(87.17)	04(10.25)	<0.0001
PT/INR, APTT	13(33.33)	0	<0.0001	17(43.58)	0	<0.001
HbA1C	01(2.56)	0	1.000	01(2.56)	0	1.000
TFT	04(10.25)	0	0.115	05(12.82)	0	0.054
Serum Electrolyte	38(97.43)	08(20.51)	<0.0001	38(97.43)	08(20.51)	<0.0001
CXR	14(35.89)	0	<0.0001	13(33.33)	0	<0.0001
ECG	16(41.02)	06(15.38)	0.022	18(46.15)	06(15.38)	0.006

**Table 4:** Comparison of the number of visits and referral before clearing patients for both unmatched and matched cohorts analyzed using Fisher’s exact test PAEC - preanaesthesia evaluation clinic, ASA-PS - the American Society of Anesthesiologists physical status class, NICE - the National Institute for Health and Care Excellence, N - total number. Group A represents routine investigation, and group B represents surgery and patient specific investigation.

PAEC visit and referral (numbers)		All patients			ASA-PS matched			NICE matched	
	Gr A [N=58]	Gr B [N=56]	Two-sided p	Gr A [N=39]	Gr B [N=39]	Two-sided p	Gr A [N=39]	Gr B [N=39]	Two-sided p
Visits for clearance									
1-visits	49(84.48)	51(91.07)	0.394	31 (79.5)	35(89.7)	0.347	29(74.4)	35(89.7)	0.138
2-visits	09(15.51)	04(7.14)	0.238	08 (20.5)	03 (7.7)	0.191	10(25.6)	03(7.7)	0.065
4-visits	0	01(1.78)	0.491	0	01 (2.6)	0.491	0	01(2.6)	0.491
Referrals									
Total	06 (10.3)	08 (14.3)	0.404	04 (10.3)	06(15.4)	0.504	05(12.8)	06(15.4)	0.748
Median (range)	0 (0-1)	0 (0-2)		0 (0-1)	0(0-1)		0(0-1)	0(0-1)	

**Table 5:** Comparison of the cost incurred by the patients of the different cohorts analysed using unpaired t-test SD - standard deviation, q3-q1 - interquartile range. Group A represents routine investigation, and group B represents surgery and patient specific investigation.

Cost / Visit	Group A [N=58]	Group B [N=56]	% Reduced	Two-sided p-value
Hospital visits - total (range)	289 (1-15)	135 (1-7)		
Hospital visits, median (q3-q1)	4 (6-3)	2 (3-1)	50.0	<0.0001
All investigations - total	40028	4330	89.18	<0.0001
All investigations, average per patient	690.13	77.32	88.88	
All investigations, mean + SD	690.13±313.9	77.32±96.8		
Travelling cost	51282	9970		
Travelling cost, average per patient	884.17	178.03	79.87	
Travelling cost, mean + SD	884.17 + 1240.1	178.03 + 126.5		<0.0001
Total cost including stay	86070	15000	82.57	
Total cost, average per patient	1483.96	267.85	81.95	
Total cost, mean + SD	1483.96±1147.1	267.85±185.6		<0.0001

**Table 6:** Comparison of number of visits and cost incurred (in Indian Rupees) ASA-PS - The American Society of Anesthesiologists physical status, NICE - The National Institute for Health and Care Excellence, SD - standard deviation, q3-q1 - interquartile range). Group A represents routine investigation, and group B represents surgery and patient specific investigation.

Parameters (Cost/ Visit)		ASA-PS matched				NICE surgical grade matched		
	Group A [N=39]	Group B [N=39]	% Reduced	Two-tailed P	Group A [N=39]	Group B [N=39]	% Reduced	Two-tailed p
Hospital Visit								
Total	193	101	47.67	<0.0001	203	95	53.2	<0.0001
Median (q3-q1)	5 (6-2)	2 (4-2)	47.67		5 (7-3)	2 (3-1)	60.0	
Range	1-15	1-7			1-15	1-7		
All Investigations								
Total	30683	3690	87.97	<0.0001	31003	3610	88.36	<0.0001
Average (per patient)	786.7	94.6	87.97		794.94	92.56		
Mean + SD	786.7±572.3	94.6±105.0			794.9±567.4	92.5±99.7		
Cost of Visit								
Total	36732	7030	80.86	0.001	41102	6840	83.36	<0.0003
Average (per patient)	941.8	180.2	80.86		1053.89	175.38	83.36	
Mean + SD)	941.8±1408.0	180.2±128.1			1053.9 ±1429.4	175.4 ± 132.4		
Cost including stay								
Total	70915	10720	84.88	<0.0001	78105	10450	86.62	<0.0001
Average (per patient)	1818.3	274.8	84.88		2002.69	267.94	86.62	
Mean + SD	1818.3±1940.7	274.8±163.7			2002.7±2012	267.9±162.48		

## DISCUSSION

The present study affirms several points. First, it proves the hypothesis that IT-based joint preoperative patient evaluation is feasible and appears to be even feasible in countries with low per-capita expenditure in the health sector. Second, the joint preoperative assessment is economically advantageous for both the patient and health care providers. The present study finding indicates that adopting the new technique reduced the number of preoperative investigations performed and reduced hospital visits by 50%. Third, surgery and patient specific investigation are adequate and do not risk case cancellation compared to the routine testing practice. It neither carried the risk of doing extra testing and intervention during the perioperative period nor affected the perioperative management and outcome. The guideline also recommends rational preoperative assessment-based investigation while avoiding costly and unnecessary tests^9^. The cost-effectiveness of routine testing is questioned by developing countries too^10^.In our study, the investigation was rationalised using a patient and surgery specific investigation protocol. Even the majority (80.9%) of the abnormal test results found in routine preoperative tests are predictable from patients' clinical condition^11^. Notably, most abnormal test results in routine preoperative tests do not change anaesthesia management^111-13^, even in oncological and older patients^14,15^. However, laboratory tests specific to the patient’s comorbid condition has an impact in changing the anaesthesia management^16^. Fourth, it reiterates that routine preoperative investigations are unnecessary and does not contribute to patient safety. The perioperative risk depends on the urgency of surgery, patient and surgery-specific risks. Therefore, patient and surgery specific preoperative assessment and investigation for risk assessment also appear to be rational approach^17^. Even routine testing in older comorbid patients undergoing minor surgeries do not contribute to patient safety^18^. Although it is agreed upon almost unanimously that routine preoperative testing is unnecessary, the implementation is lacking in clinical practice due to various reasons^7^. The adopted method can bring all related health-service provider specialists into one platform, thereby nullifying the concept that other specialities require the investigation.

Although anaesthesia information management and hospital information management services (HIMS) are available in many developed countries, their availability is scarce in developing and third world countries. It is also being observed that these technology-based patient management solutions mostly do not work on the concept of joint preoperative assessment before ordering preoperative investigations and referrals in clinical practice. Many hospital set-ups may not even have enough budgetary allocations to afford a fully-fledged, advanced, and costly software. The present software developed locally is cheap. It incorporates outpatient data related to patients' disease, clinical findings, sections for ordering and documenting laboratory tests, referral notes, anaesthesia-related clinical examinations, preoperative advice sections, intraoperative and postoperative events log.

As the computers are readily available hardware components in most of the hospitals in the present time, the software can be installed in different computers, and data can be accessed from any computer in the hospital connected through local area networks or through cloud-computing based technology^19,20^. While cloud computing-based technology needs access to the cloud (data storage and retrievement through the internet) costs extra few dollars per year, local area network based locally developed network has also been found to be economically cheap^19^. Furthermore, if such an anaesthesia module is not available in the existing HIMS, it can be incorporated into it. The study findings of saving cost for even the ASA-PS and the NICE invasive grade-matched group indicate that the minimal investment incurred by adopting the new technique can contribute to the nation's economy without compromising the patient care quality and safety.

In our project, we faced a few hurdles, especially in the implementation of the workflow. Although it was planned that the patients' data entry would start right from the time the patient visits the surgery outpatient department (OPD), it was not possible, and it was started in the PAEC OPD. It did not impact the study objective as the patient attended the PAEC on the same day, and advice for investigation or referral was also provided on the same day.

Investigation reports were also manually entered by the research assistant on re-visit or the day of surgery. These hurdles can be easily overcome by integrating the software in HIMS and automatically retrieving data from laboratories. Further, the medical record department or concerned department can maintain a central registry, and once the patient is approved for elective surgery, they can be given a date for surgery and informed over telephone/email. It will also save one more working day for the patients who often need to visit surgery OPD/clinic for scheduling the surgery in many centres/set-ups.

The study also included patients mostly from one speciality. It was done for smoother conductance of the project, as the change of routine practice required both surgeons and anaesthesiologists to agree upon the protocol. We included only ASA-PS I to III patients, and hence the result may not be extrapolated for ASA-IV/V patients. Routine preoperative viral screening tests are also not beyond controversy^21^. We excluded these tests as it was done in all cases as per the institute’s protocol. Furthermore, the cost calculation was performed for our institution, where the investigation costs are subsidised. The cost savings are likely to be higher in other centres without subsidies. In addition, the present study was conducted in a single apex level teaching institute, which might have impacted the number of ordered tests^22^.Moreover, only a few patients required to stay in a hotel/lodge, which may not represent the centres that cater to only local patients or a high number of out-station patients. Therefore, actual cost savings are likely to vary for different set-ups. Nevertheless, the conclusion derived is likely to hold even for set-ups/healthcare systems where a patient does not need to pay, as the number of tests ordered was also low, indicating that the service provider will require to do fewer tests, thereby saving costs.

## CONCLUSION

IT-based joint preoperative assessment is feasible through locally developed software with minimal cost. It can help in the practical application of patient and surgery specific preoperative investigation, reduce the number of tests, hospital visit, and cost without adversely affecting the perioperative outcome. The application of the modified method will help in cost-effective, yet good quality and safe perioperative healthcare delivery and it will be of benefit for the public from both a service and an economic perspective.

## Key Points

**◊** *Despite the evidence-based negative recommendations, routine preoperative investigations are widespread*

**◊** *Both healthcare providers and policymakers are searching for strategies that can reduce unnecessary investigations without compromising service quality and patient safety*

**◊***The public is the consumer of healthcare. Cost-effective, yet good quality and safe perioperative healthcare delivery will benefit the public from both a service and economic perspective*.
